# Effect of β,β-Dimethylacrylshikonin on Inhibition of Human Colorectal Cancer Cell Growth *in Vitro* and *in Vivo*

**DOI:** 10.3390/ijms13079184

**Published:** 2012-07-23

**Authors:** Yingying Fan, Shaoju Jin, Jun He, Zhenjun Shao, Jiao Yan, Ting Feng, Hong Li

**Affiliations:** 1 Key Laboratory of Birth Defects and Obstetric & Gynecologic and Pediatric Disease of Ministry of Education, West China Second University Hospital, Sichuan University, Chengdu, 610041, Sichuan, China; E-Mails: fyy1119@yahoo.com.cn (Y.F.); shaozhenjun2006@163.com (Z.S.); fengting0107@yahoo.com.cn (T.F.); 2 Department of Pharmacology, School of Pharmacy, Ningxia Medical University, Yinchuan, 750004, Ningxia, China; E-Mail: jinshaoju@163.com; 3 Department of Radiology, West China University Hospital, Sichuan University, Chengdu, 610041, Sichuan, China; E-Mail: hejun19850111@163.com; 4 Chengdu General Military Hospital, Chengdu, 610083, Sichuan, China; E-Mail: jocye0413@163.com

**Keywords:** β,β-dimethylacrylshikonin, apoptosis, anti-tumor, colorectal cancer

## Abstract

In traditional Chinese medicine, shikonin and its derivatives, has been used in East Asia for several years for the prevention and treatment of several diseases, including cancer. We previously identified that β,β-dimethylacrylshikonin (DA) could inhibit hepatocellular carcinoma growth. In the present study, we investigated the inhibitory effects of DA on human colorectal cancer (CRC) cell line HCT-116 *in vitro* and *in vivo*. A viability assay showed that DA could inhibit tumor cell growth in a time- and dose-dependent manner. Flow cytometry showed that DA blocks the cell cycle at G^0^/G^1^ phase. Western blotting results demonstrated that the induction of apoptosis by DA correlated with the induction of pro-apoptotic proteins Bax, and Bid, and a decrease in the expression of anti-apoptotic proteins Bcl-2 and Bcl-xl. Furthermore, treatment of HCT-116 bearing nude mice with DA significantly retarded the growth of xenografts. Consistent with the results *in vitro*, the DA-mediated suppression of HCT-116 xenografts correlated with Bax and Bcl-2. Taken together, these results suggest that DA could be a novel and promising approach to the treatment of CRC.

## 1. Introduction

Although advances have been made in clinical medicine, no effective treatment modalities are available for patients with advanced or metastatic tumors [1]. Colorectal cancer (CRC) is one of the most common causes of cancer-related mortalities in both China and the Western world [2]. Moreover, conventional treatments such as chemotherapy and radiotherapy can increase the risk of potential complications. Hence, various natural phytocompounds have been screened and investigated extensively as potential anti-cancer agents [3].

**Figure 1:**
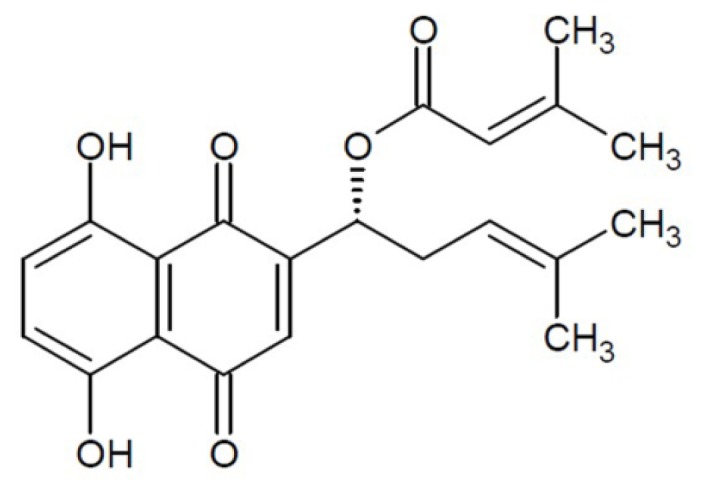
Molecular structure of β,β-dimethylacrylshikonin (DA).

β,β-dimethylacrylshikonin (DA) is isolated mainly from the roots of *Lithospermum erythrorhizon* that belong to the Boraginaceae family (Figure 1) [4]. The present study and other reports have shown that shikonin and its derivatives possess antitumor activity in a variety of human cancers, including hepatocellular carcinoma and gastric carcinoma [5,6]. Previous studies have also shown that shikonin can possess multiple pharmacological properties such as anti-inflammatory, antioxidant, anti-platelet, and anti-atherosclerotic effects [7–10]. However, little is known about the inhibitory effect of DA on CRC, especially *in vivo*.

In the present study, we investigated the efficacy of DA in CRC cells and nude mice tumor models with the aim of developing a novel CRC treatment approach.

## 2. Results and Discussion

### 2.1. Effect of DA on Inhibition of Cell Growth *in Vitro*

**Figure 2:**
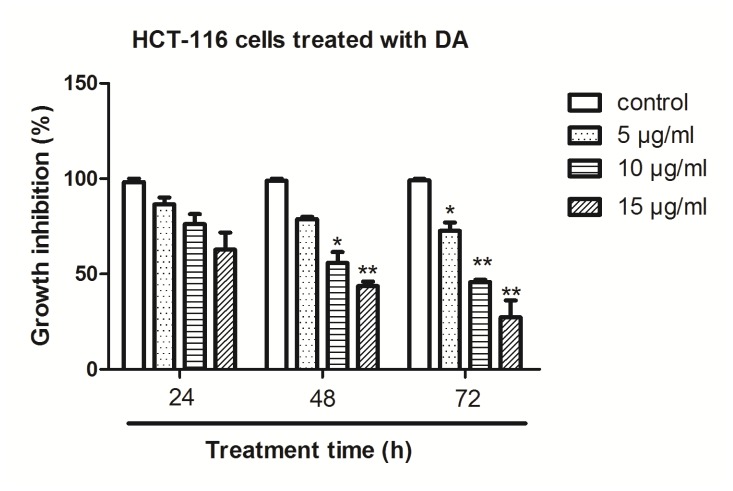
Effect of DA on the inhibition of HCT-116 cell proliferation *in vitro*. Cells were treated with DA (5, 10, 15 μg/mL) for 24, 48 and 72 h. The levels of cell viability were measured by MTT method compared to untreated cells. Results represent the means ± SD from six independent experiments. *p < 0.05 and **p < 0.01, compared with the control.

HCT-116 cells were treated with DA and the cell viability was determined by MTT assay. The treatment of HCT-116 cells for 24–72 h with 5, 10 and 15 μg/mL of DA resulted in cell growth inhibition in a dose- and time-dependent manner (Figure 2). To confirm cell growth inhibition, we also conducted a cell proliferation assay using the BrdU labeling and Detection Kit (Roche, Indianapolis, IN); we found similar results as in the MTT assay using this method (data not shown).

### 2.2. DA-Induced Cell Cycle Arrest in HCT-116 Cells

**Figure 3:**
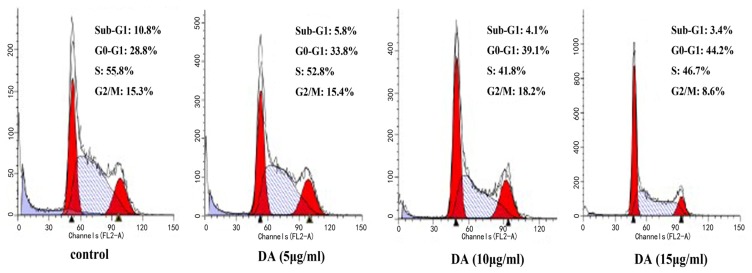
Effect of DA on cell cycle distribution. HCT-116 cells were harvested for cell cycle analysis using propidium iodide staining after incubated for 15 min. *X* axis, DNA content; *Y* axis, cell number.

To investigate the effect of DA on cell cycle distribution of HCT-116 cells, the DNA content was analyzed by flow cytometry. As shown in Figure 3, it indicated that DA causes a significant increase in the percentage cells in the G^0^/G^1^ phase of the cell cycle in a dose-dependent manner.

### 2.3. DA Induced Apoptosis in HCT-116 Cells

**Figure 4:**
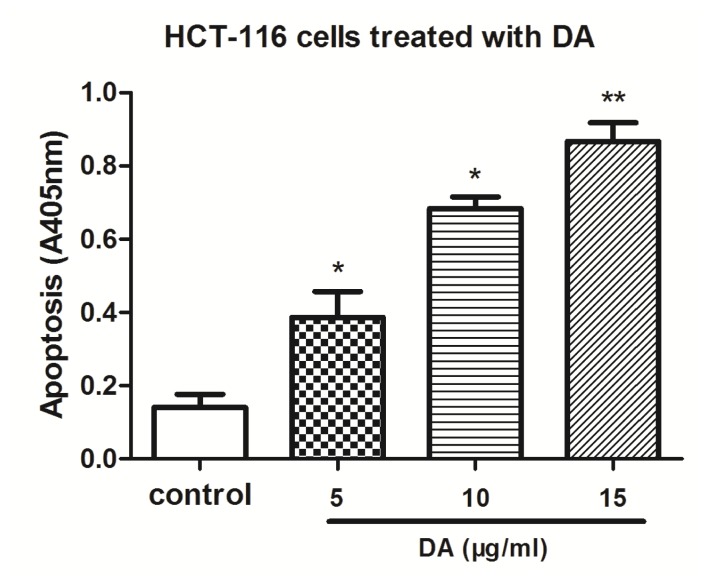
Effect of DA on cell apoptosis. Cell death assay for measuring apoptosis induced by DA were cultured in RPMI-1640 containing 5% FBS and exposed to different doses of DA for 48 h. Apoptosis was measured by Histone/DNA ELISA Kit. Values are reported as means ± SD. *p < 0.05 and **p < 0.01, compared with the control.

HCT-116 cells were treated with 5, 10 and 15 μg/mL of DA for 48 h. After treatment, the degree of apoptosis was measured. The induction of apoptosis was found to be dose-dependent (Figure 4). These results provided convincing data showing that DA could induce apoptosis in HCT-116 cells.

### 2.4. DA Regulates the Expression of Bcl-2 Family Proteins

**Figure 5:**
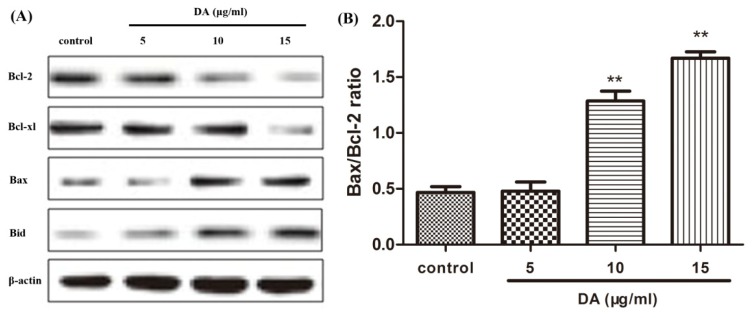
DA regulates Bcl-2 family protein expression. (**A**) Cells were treated with different doses of DA for 48 h. Bcl-2, Bcl-xl, Bax and Bid were determined by Western blotting using specific antibodies. (**B**) A densitometric analysis was used to quantify the levels of Bax and Bcl-2 to evaluate the effect of DA on the Bax/Bcl-2 ratio. All results were obtained from three independent experiments. *p < 0.05 and **p < 0.01, compared with the control.

The Bcl-2 family plays a major role in the regulation of apoptosis by functioning as promoters or inhibitors of cell death. We therefore examined the expression of Bcl-2 family proteins in DA-treated HCT-116 cells in a dose-dependent manner. As shown in Figure 5A, DA suppresses the expression of anti-apoptotic proteins such as Bcl-2 and Bcl-xl and moderately increases the expression levels of pro-apoptotic proteins such as Bax and Bid. In addition, the ratio of Bax and Bcl-2 was measured by quantification of bands. The results indicated that DA treatment induces a dose-dependent increase in the Bax/Bcl-2 ratio in HCT-116 cells (Figure 5B).

### 2.5. Effect of DA on Inhibition of HCT-116 Xenografts in Nude Mice

**Figure 6:**
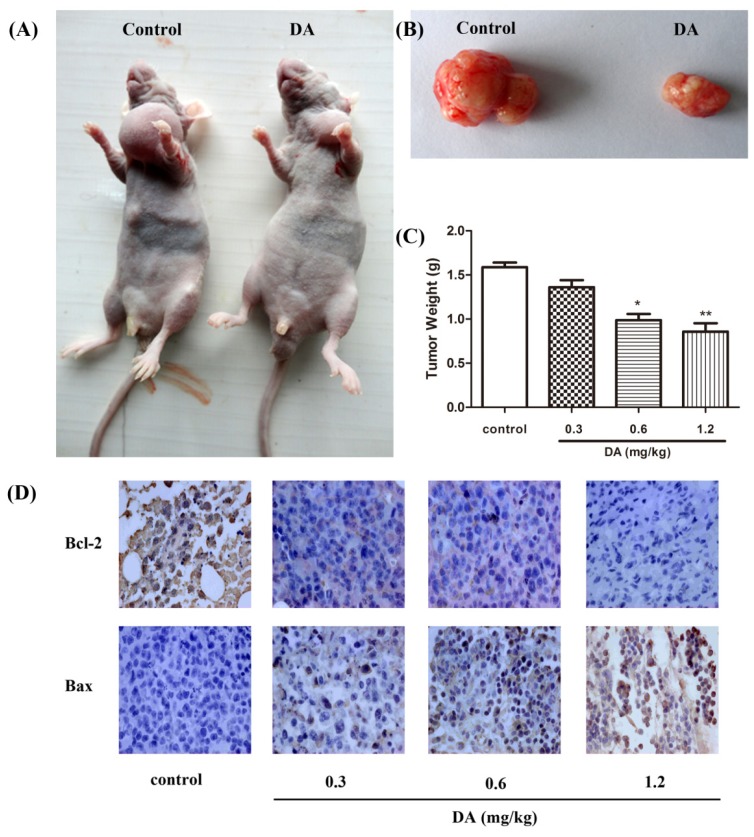
Effect of DA on tumor growth and the expression of Bcl-2 family *in vivo*. HCT-116 xenografts were generated by inoculating cells subcutaneously (s.c.) in nude mice. The control group was injected with an equal amount of saline, and the experimental group was injected with different doses of DA. (**A**, **B**) Photograph of the tumor in the control group and the DA-treated group. (**C**) Tumor weight at time of sacrifice for different treated groups. *p < 0.05 and **p < 0.01, compared with the control. (**D**) Immunohistochemical staining for Bcl-2 and Bax in HCT-116 xenograft tumor.

To determine whether systemic therapy with DA could stunt tumor growth in animals, we established HCT-116 xenografts in nude mice. As shown in Figure 6C, DA treatment significantly inhibited tumor growth compared with untreated control. In addition, no toxicity judged by parallel monitoring of the body weight was observed in DA-treated mice. Immunohistochemical staining showed that the expression of Bcl-2 was down-regulated in the DA-treated group, whereas, the expression of Bax was markedly higher in the DA-treated group (Figure 6D).

## 3. Materials and Methods

### 3.1. Chemical Reagents

DA was obtained from Huakang Pharmaceutical Company (Deyang, China) and the purity was determined by high-performance liquid chromatography.

### 3.2. Cell Lines and Culture

Human CRC cell lines HCT-116 were obtained from the Shanghai Institute of Cell Biology, Chinese Academy of Sciences. The cells were cultured in RPMI-1640 media (Invitrogen, Carlsbad, CA) supplemented with 10% fetal bovine serum (Minhai Biotech, Lanzhou, China) and antibiotics (100 U/mL penicillin, 100 mg/mL streptomycin). All cells were grown in 5% CO^2^ at 37 °C and were sub cultured at an initial density of 1 × 10^5^ cells/mL every three days. All experiments were performed with cells in the logarithmic phase of growth.

### 3.3. Cell Proliferation Assay

The cells (5 × 10^3^) were seeded in a 96-well plate and subsequently treated with DA (2.5, 5, 10 μg/mL) for 12, 24 and 48 h. Twenty micro liters of MTT (5 mg/mL) solution (Sigma, St Louis, MO) were added to each well at 37 °C for four hours. After removing the media, 150 μL of dimethylsulfoxide (DMSO) were added and left for 20 min at room temperature to dissolve the formazan crystals in a shaker. The absorbance at 570 nm was measured in a micro plate reader (Bio-Rad, Hercules CA).

### 3.4. Cell Cycle Analysis

To estimate the proportion of cells in the different phases of the cell cycle, the cell DNA contents were measured using flow cytometry. HCT-116 cells plated on 20-cm^2^ tissue culture flasks were collected at 24 h after the incubation with vehicle and DA. Then, the cells were fixed gently with 70% ethanol in ice and were then resuspended in PBS containing 50 μg/mL PI (Sigma, St Louis, MO) and 0.1 mg/mL RNase (Sigma, St Louis, MO). After 30 min at 37 °C, the cells were analyzed with a flow-cytometer and the cell cycle was determined. Experiments were performed in triplicate and repeated three times.

### 3.5. Histone/DNA ELISA for Detection of Apoptosis

The cell death detection ELISA kit (Roche, Indianapolis, IN) was used for assessing apoptosis according to the manufacturer’s instructions. Briefly, cells were treated with DA for 48 h. After treatment, the cells were lysed and the cell lysates were overlaid and incubated in a micro titer plate modules coated with anti-histone antibody for detection of apoptosis.

### 3.6. Western Blotting

Western blotting was performed as we have previously described [6]. Briefly, equal amounts of protein were separated by 12% SDS-PAGE and transferred into a PVDF membrane (Bio-Rad, Hercules, CA). After blocking with 5% non-fat milk at 4 °C overnight, the membranes were incubated with specific primary antibodies (Santa Cruz, Calif, USA) at room temperature for 2 h. Washing the membranes with TBST three times, and then incubated with horseradish peroxidase (HRP)-conjugated antibody (Santa Cruz, Calif, USA) at room temperature for 1 h. The signal was detected using an enhanced chemiluminescence kit (Boster, Wuhan, China).

### 3.7. Human Tumor Xenografts in Nude Mice

Male nude mice (4- to 5-week old) were obtained from the Sichuan University Animal Center. Mice were housed in temperature-controlled rooms with a 12-h alternating light-dark cycle. HCT-116 cells (1 × 10^7^ cells/mL, 0.2 mL/mouse) were injected subcutaneously into the upper left flank region of mice. The mice were randomly divided into four groups (*n* = 6 per group). Mice in each group were treated daily with DA (0.3, 0.6, 1.2 mg/kg) or the same volume of saline as a negative control group by intraperitoneal administration every alternate day from day one. The dose of DA was based on our previous work that did not show any cytotoxic effect in normal mice. All the mice were sacrificed on day 13 after inoculation with HCT-116 cells. The tumor was measured for largest (a) and smallest (b) diameters, and the tumor volume was calculated as *V* = a × b^2^/2. Tumor inhibitory rates were calculated with the following formula: tumor inhibitory rate (%) = 1 − (tumor weight of treated group/tumor weight of control group) × 100%. Guidelines for the humane treatment of animals were followed as approved by the Sichuan University.

### 3.8. Immunohistochemical Staining

DA-embedded nude mouse xenograft tissues were deparaffinized in xylene and rehydrated in graded alcohol. Endogenous peroxidase was blocked using hydrogen peroxide for 20 min. The slides were incubated with primary antibody of Bcl-2 and Bax overnight at 4 °C, and then incubated with a second antibody for 2 h at room temperature. Binding streptavidin-HRP was 20 min at RT. Staining time of DAB (Boster, Wuhan, China) solution depended on the sample condition. The stained slides were visualized on a microscope. Images were captured with an attached camera linked to a computer.

### 3.9. Statistical Analysis

The results are expressed as the mean ± SD. Multiple comparisons were performed by one-way ANOVA followed by Duncan’s post-hoc test using SPSS 18.0 (Chicago, IL). A value of *p* < 0.05 was considered statistically significant.

## 4. Conclusions

Many compounds extracted from *Lithospermum erythrorhizon* have been proven to have antitumor activity against a number of cancer cells, and DA is one of the most effective agents. Previous studies demonstrated that shikonin and its derivatives exerted inhibition effects on several tumor cell lines, including human premyelocytic leukemia cell line HL-60, human breast cancer cell line MCF-7, human oral squamous cell carcinoma cell line Tca-8113, human lung adenocarcinoma cell line A549 and so on [11–14]. The antitumor effects of shikonin had also been confirmed with *in vivo* animal experiments such as S180 sarcoma model and Lewis lung carcinoma model [15,16]. However, little is known about the anticancer activity of DA on human CRC cells, especially *in vivo*.

In the present study, we found that DA inhibited the proliferation of human CRC cell line in a dose- and time-dependent manner *in vitro*. Moreover, DA could suppress the tumor growth of HCT-116 xenografts. Generally, tumorigenesis and tumor progression are strongly associated with apoptosis.

We firstly analyzed the effect of DA in human colorectal cancer cell line HCT-116. Consistent with our previous studies, DA caused significant inhibition of tumor growth. Flow cytometric analysis of the effects of DA on the cell cycle in treated HCT-116 cells revealed a dose-dependent decrease in cell proliferation and a concomitant accumulation of cells in G^0^/G^1^ phase. We also found that DA could induce apoptosis, a finding confirmed by Histone/DNA ELISA kit.

We also investigated the underlying mechanisms for the apoptosis induction of CRC cells. Apoptosis, or programmed cell death, is an essential physiological process that is required for normal development and maintenance of tissue homeostasis [17]. Initiation of apoptosis appears to be an important mechanism of antitumor agents used in chemotherapy. Members of the Bcl-2 family are important regulators in the apoptotic pathway with individual members that promote and suppress apoptosis. The proteins of the Bcl-2 family include both pro- and anti-apoptotic members that elicit opposing effects on mitochondria. Enhancement of pro-apoptotic Bax over Bcl-2 proteins can enhance the permeability of the mitochondrial membrane, which in return results in the release of apoptogenic factors. Repression of anti-apoptotic members of this family, including Bcl-2 and Bcl-xl, preserves the integrity of the mitochondria [18–20]. In our work, it was found that DA down-regulated the Bcl-2 and Bcl-xl expression, while concomitantly up-regulating the Bax and Bid expression, and the ratio of Bax/Bcl-2 increased correspondingly, which could be an important mechanism contributing to the induction of tumor cell apoptosis by DA.

Next, we conducted experiments designed to test the potential for DA to exert protective effects against colorectal cancer *in vivo*. Our results indicated that treatment with DA could inhibit the growth of the HCT-116 xenografts in a dose-dependent manner. Consistent with the results *in vitro*, the immunohistochemical staining showed that DA decreased the expression of Bcl-2 and increased the expression of Bax.

In summary, we presented experimental evidence, which strongly supports the antitumor effects of DA in CRC *in vitro* and *in vivo* by inducing apoptosis involved in down-regulation of Bcl-2 expression and up-regulation of Bax expression. Our study suggests that DA represents a promising novel agent that should be developed for the treatment of CRC.
